# MiR-1539 and Its Potential Role as a Novel Biomarker for Colorectal Cancer

**DOI:** 10.3389/fonc.2020.531244

**Published:** 2021-02-18

**Authors:** Xueyang Cui, Zhi Lv, Hanxi Ding, Chengzhong Xing, Yuan Yuan

**Affiliations:** ^1^ Tumor Etiology and Screening Department of Cancer Institute and General Surgery, The First Hospital of China Medical University, Shenyang, China; ^2^ Key Laboratory of Cancer Etiology and Prevention in Liaoning Education Department, The First Hospital of China Medical University, Shenyang, China; ^3^ Key Laboratory of GI Cancer Etiology and Prevention in Liaoning Province, The First Hospital of China Medical University, Shenyang, China

**Keywords:** miR-1539, exosome, serum, tissue, biomarker, colorectal cancer

## Abstract

**Purpose:**

We investigated microRNA (miR) 1539 as a potential biomarker for predicting the risk and pathobiological behavior of colorectal cancer (CRC).

**Methods:**

Our strategy consisted of analyzing 100 serum samples from 51 CRC patients, 49 healthy controls (HCs), and another 56 CRC tissue and matched normal adjacent to tumor (NAT) samples. The relative expression levels of miR-1539 in exosomes, serum and tissues were detected and compared in the different groups, using reverse transcription-polymerase chain reaction (RT-qPCR). The diagnostic value and potential function of miR-1539 were investigated using clinicopathological data combined with bioinformatics analysis.

**Results:**

MiR-1539 expression was significantly up-regulated in exosomes (*p* = 0.003) and cancer tissue (*p* < 0.001) from CRC patients. MiR-1539 expression levels in serum varied according to different tumor sites (right-sided vs. left-sided, *p* = 0.047; left-side CRC vs. HCs, *p* = 0.031). In terms of diagnostic efficacy, miR-1539 expression in exosomes may help distinguish CRC cases from HCs with a sensitivity of 92.2%, and miR-1539 expression in serum may improve the specificity to 96.6% for left-sided CRC diagnosis. When combined with clinicopathological data, serum miR-1539 levels were positively associated with vascular endothelial growth factor (VEGF) expression (*p* = 0.028), whilst levels in CRC tissue were positively associated with increased Ki-67 levels (*p* = 0.035). Poorer pathologic differentiation was potentially related to an increased tendency of miR-1539 expression in CRC tissue (*p* = 0.071). Based on our bioinformatics analysis, miR-1539 may have a significant mechanistic influence on CRC genesis and progression.

**Conclusions:**

Circulating or tissue based miR-1539 may be used as a novel potential biomarker for CRC screening, and a predictor of poor clinicopathological behavior in tumors.

## Introduction

According to Global Cancer Observatory (GLOBOCAN) 2018 estimates, colorectal cancer (CRC) is the fourth most common malignancy, and the second leading cause of cancer-related death worldwide (1). Despite the fact that survival outcomes for CRC have substantially improved in recent decades, the incidence and mortality of CRC is still rapidly rising in developing and middle-income countries (2). Therefore, the early detection and identification of efficient non-invasive biomarkers is crucial for CRC diagnosis, and reducing the burden of CRC mortality.

MicroRNAs (miRNAs) are a series of mature small non-coding RNAs (typically 21–25 nucleotides), first discovered in 1993 (3, 4). The molecules are postulated to assist in the regulation of cancer-related gene expression in both cells and tissues, by directly targeting mRNAs or inhibiting protein biosynthesis (4, 5). As potential biomarkers, miRNAs can be detected in body fluids or tumor tissues. They have been implicated in the diagnosis and prognosis of various solid tumors, thanks to their role in cancer genesis and progression (6–9). Notably, circulating miRNAs are robust and stable (10). They are packed into extracellular vesicles (particularly exosomes) or bind to protein complexes, which makes them early stage non-invasive indicators of disease (10–15). To date, several miRNA detection methods have been proposed; quantitative reverse-transcriptase polymerase chain reaction (RT-qPCR), next generation sequencing, microarrays, *in situ* hybridization and nanopore technology, etc (16, 17). RT-qPCR is the most common and convenient strategy; it is consistently reliable and has been a gold standard approach for quantifying circulating miRNAs (17). While the diagnostic value of several miRNAs has been previously confirmed (6–9), many miRNAs require exploration in terms of function and potential as biomarkers in various cancers.

MiR-1539 is 21 nucleotides long, to date, a few articles have been published (18–20). He et al. observed that miR-1539 was inhibited by brain-derived neurotrophic factor, which stimulated angiogenesis and promoted endothelial cell survival (18). Ayaz et al. indicated that miR-1539 overexpression was putatively involved in the pathological process of senile macular degeneration, with oxidative stress (19), whereas Veija et al. reported that miR-1539 was associated with polyomavirus-positive Merkel cell carcinoma (20). However, the function of miR-1539 and its relationship with disease, especially cancer, remains unknown. Previously, we discovered that miR-1539 was differentially expressed in CRC cases and healthy controls (HCs), based on serum exosome miRNA microarray screening (unpublished results). These data suggest that miR-1539 may be associated with CRC, therefore the role of miR-1539 in CRC development requires further clarification.

In this study, we focused on the association and diagnostic value of miR-1539 expression levels with CRC risk. This strategy was based on a multidimensional case-control study approach using exosomes, serum, and tissue samples.

## Materials and Methods

### Study Subjects

In total, 100 serum samples comprising 51 CRC cases and 49 healthy controls (HCs), and another 56 pairs of cancer tissues and matched normal adjacent tissue (NAT) from CRC patients were collected from the First Affiliated Hospital of China Medical University between August 2018 and July 2019. All CRC patients underwent preoperative colonoscopy, and were diagnosed with adenocarcinoma using histopathology, without radiotherapy or chemotherapy. Their clinicopathological data included tumor size, differentiation, TNM stage, angiolymphatic invasion, perineural invasion, and tumor deposits. In addition, information on Ki-67, p53, epidermal growth factor receptor (EGFR), and vascular endothelial growth factor (VEGF) by immunohistochemistry (IHC) were also collected. The HCs group was selected from individuals seeking physical examination without malignancy. The study was approved by the ethics committee of the First Affiliated Hospital of China Medical University and written informed consent as prescribed by the Declaration of Helsinki was collected for each participant.

### Sample Collection

Peripheral venous blood (5 ml) from all subjects was collected using BD Vacutainer™ Flashback Blood Collection Needles (BD SST™, ref. 301746, NJ, USA), and golden top BD Vacutainer™ serum blood collection tubes (BD SST™, ref. 367986, NJ, USA), with silica clot activator, polymer gel and silicone-coated interior. Whole blood samples were maintained at room temperature for 30 min to ensure complete coagulation. Then, serum was extracted by centrifugation at 3,000×g for 10 min, and immediately stored at -80°C until required. Tissue samples were obtained immediately after resection and stored at −80°C.

### Serum Exosomal RNA Isolation and Exosome Identification

Exosomes were isolated from serum using ExoQuick™ exosome precipitation solution (System Biosciences, Palo Alto, CA), according to manufacturer’s instructions. Exosomal pellets were re-suspended in phosphate buffer saline (PBS) and stored at -80°C. Serum exosomal RNA was extracted using the SeraMir™ Exosome RNA Amplification Kit (System Biosciences, Palo Alto, CA), according to manufacturer’s instructions. Briefly, 120 µl ExoQuick solution was gently mixed with 500 µl serum. After incubation at 4°C for 30 min, samples were centrifuged at 13,000 rpm for 2 min to generate pellets. The supernatant was discarded and 350 µl lysis buffer was immediately added. After purification, the RNA was typically eluted in 30 µl from serum exosomes.

To verify that pellets were exosomes, we performed transmission electron microscopy (TEM), nanoparticle tracking analysis (NTA) and western blotting.

### Transmission Electron Microscopy (TEM)

Exosome suspensions were placed on a formvar carbon-containing grid for 1 min, and excess liquid was aspirated with clean filter paper. Then phosphotungstic acidoxalate was added for 1 min, and again, excess liquid was removed. The grid was dried for 10 min at room temperature, followed by exosome visualization on a JEM-2100 Plus TEM (JEOL, Tokyo, Japan) at 100 kV, to capture images (magnification: 50,000×).

### Nanoparticle Tracking Analysis (NTA)

For NTA measurements, we used a ZetaView PMX 110 (Particle Metrix, Meerbusch, Germany) instrument to determine exosome size distribution and concentration. The exosomal concentration was determined at 1.8×10^11^ particles/ml, therefore we diluted this solution 5,000-fold to achieve optimal conditions for NTA measurements.

### Western Blotting

Total exosomal proteins were harvested in RIPA buffer (Solarbio, Beijing, China), followed by sonication and centrifugation at 13,000×g for 30 min. Proteins were then prepared for western blotting. Denatured proteins were electrophoretically separated using 10% sodium dodecyl sulfate-polyacrylamide gel electrophoresis (SDS-PAGE), and then transferred to polyvinylidene fluoride (PVDF) membranes. The membranes were blocked in 5% skimmed milk for 1 h at room temperature. Membranes were incubated overnight with primary antibodies at 4°C. The next day, membranes were washed in tris-buffered saline with 0.1% tween 20 (TBST: 20 mM Tris, 150 mM NaCl, 0.1% Tween 20, adjust pH to 7.4–7.6) and followed by incubation with secondary antibodies for 1 h at room temperature. All the aforementioned antibodies were from Abcam (Danvers, MA), including primary antibodies anti-CD9 1:2000 (ab92726), anti-TSG101 1:2000 (ab125011), and secondary antibodies goat anti-rabbit IgG 1:5000 (ab205718). After incubation and washing, the target bands were visualized by enhanced chemiluminescence (ECL) reagent (BeyoECL Moon, Shanghai, China) in accordance with the manufacturer’s instructions.

### Serum RNA Isolation

Serum RNA was extracted from 200 μl volume using miRcute Serum/Plasma miRNA Isolation Kit (Tiangen, Beijing, China) in accordance with user manual. Each sample was diluted in 30 μl RNase-free water and stored at -80°C until use. Optical densities at 260/280 nm ranged between 1.45–1.93 (NanoDrop, Thermo Scientific), with RNA yields ranging between 129–393 ng.

### Tissue RNA Isolation

Total RNA of 10 mg cancer tissue and NAT samples was isolated using Trizol Universal reagent (Tiangen, Beijing, China) according to the manufacturer’s protocol. Each sample was dissolved in 80 μl RNase-free water and stored at -80°C. Total RNA was quantified by NanoDrop (Thermo Scientific) with optical densities at 260/280 nm ranged between 1.67–1.98.

### MiR-1539 Detection by RT-qPCR

MiR-1539 cDNAs from exosomes, serum, and tissue were synthesized by miRcute Plus miRNA First-Strand cDNA Kit (Tiangen, Beijing, China), with reaction conditions as followed: 42°C for 60 min and subsequently 95°C for 3 min. Eight μl RNA derived from exosomes or serum and 2 μg tissue-based RNA were reverse-transcribed to cDNA. RT-qPCR was performed to detect the relative expression levels of miRNAs by using miRcute Plus miRNA qPCR Kit (SYBR Green, Tiangen, Beijing, China) within a 20 μl volume system. Two μl cDNA was added in each reaction. The reaction procedure was as follows: 95°C for 15 min, 45 cycles of 94°C for 20 s and 60°C for 34 s. U6 was performed as endogenous control to calculate miRNA relative expression by 2^-△Ct^ method in serum and tissue. △Ct was calculated as follows: △Ct = Ct (miR-1539)–Ct (U6). Melting curve was used to evaluate the specificity of PCR products. The primer sequence of miR-1539 was 5’-GCG TCC TGC GCG TCC CAG-3’.

### Evaluation of Diagnostic Efficacy Using ROC Curves

We conducted receiver operating characteristic (ROC) curves to evaluate the diagnostic efficacy of exosome-based and serum-based miR-1539 expression levels, between CRC and HCs groups. The area under the ROC curve (AUC) was used to assess the diagnostic efficacy in terms of sensitivity and specificity.

### Target Genes Prediction of miR-1539 by Bioinformatics Analysis and Validation by RT-qPCR

The target genes of miR-1539 were predicted by synthesis analysis of public bioinformatics algorithms miRWalk (http://mirwalk.umm.uni-heidelberg.de/) and TargetScan (http://www.targetscan.org/) datasets. We took the intersection of two databases, and common shared genes were selected for subsequent analyses. Gene Oncology (GO) analysis was performed to describe gene function in three major items: biological process, cellular component, and molecular function. Hypergeometric distribution algorithm (21) was used to evaluate the significance of differential gene enrichment in each GO item. Related biological pathways were explored by Kyoto Encyclopedia of Genes and Genomes (KEGG). Both GO and KEGG analyses were available in online tool DAVID (https://david.ncifcrf.gov).

To further confirm potential target genes, 32 pairs of CRC tissue with NAT samples were performed by RT-qPCR. Total RNA was converted into cDNA using PrimeScript RT Master Mix (Perfect Real Time, TaKaRa, Japan) with reaction conditions as followed: 37°C for 15 min and 85°C for 5 s. Relative expression levels of target mRNAs were conducted by TB Green Premix EX Taq II (Tli RNaseH Plus, TaKaRa, Japan) with following system: 95°C for 15 min, 45 cycles of 95°C for 10 s, 56°C for 20 s, and 72°C for 30 s. β-actin was regarded as endogenous reference control to evaluate target mRNA relative expression by 2^-△Ct^ method. △Ct was calculated as follows: △Ct = Ct (mRNA candidate)–Ct (β-actin). The primer sequences were tabulated (**Supplemental Table 1**).

### Statistical Analysis

Student t-test and chi-square test were used to estimate the difference of miR-1539 expression levels between groups in serum or tissue. Mann-Whitney U test was adopted when the data did not accord with normal distribution. All analyses were conducted using IBM SPSS Statistics software version 20.0 (USA) and GraphPad Prism 7.0 (USA). All the *p* values were set as 0.05 for statistical significance (*p < 0.05, ** *p* < 0.01, *** *p* < 0.001).

## Results

### Basic Characteristics of Study Participants

One hundred serum samples were used in this study, comprising 49 HCs and 51 CRC cases. No significant differences were observed in terms of age and sex between the groups (*p* > 0.05). In addition, we collected another 56 pairs of cancer and NAT tissues from CRC specimens. The demographic characteristics of all subjects are shown (**Table 1**).

**Table 1:** The baseline of the subjects.

	Serum				Tissue
	CRC (n = 51)	HCs (n = 49)	*p* value		CRC (n = 56)
Age, mean ± SD	58.92 ± 11.39	55.71 ± 7.67	0.101		61.95 ± 11.30
Sex			0.814		
Male, n (%)	29(56.9)	29(59.2)			29(51.8)
Female, n (%)	22(43.1)	20(40.8)			27(48.2)

CRC, colorectal cancer; HCs, healthy controls.

### Exosomal miR-1539 Expression Levels in CRC and Correlations With Pathological Parameters

**Figure 1:**
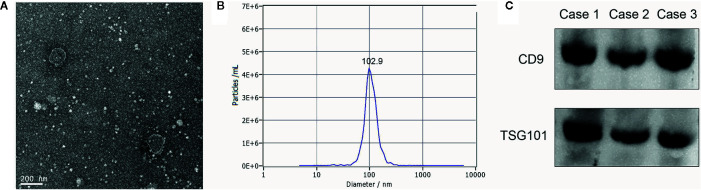
Characterization of serum exosomes **(A)** The morphology and size of exosomes was described by TEM, **(B)** The size and distribution of exosomes were determined using NTA, with 102.9 nm in diameter on average, **(C)** Western blot: analysis for typical exosome markers, CD9 and TSG101, which are enriched in exosomes.

**Figure 2:**
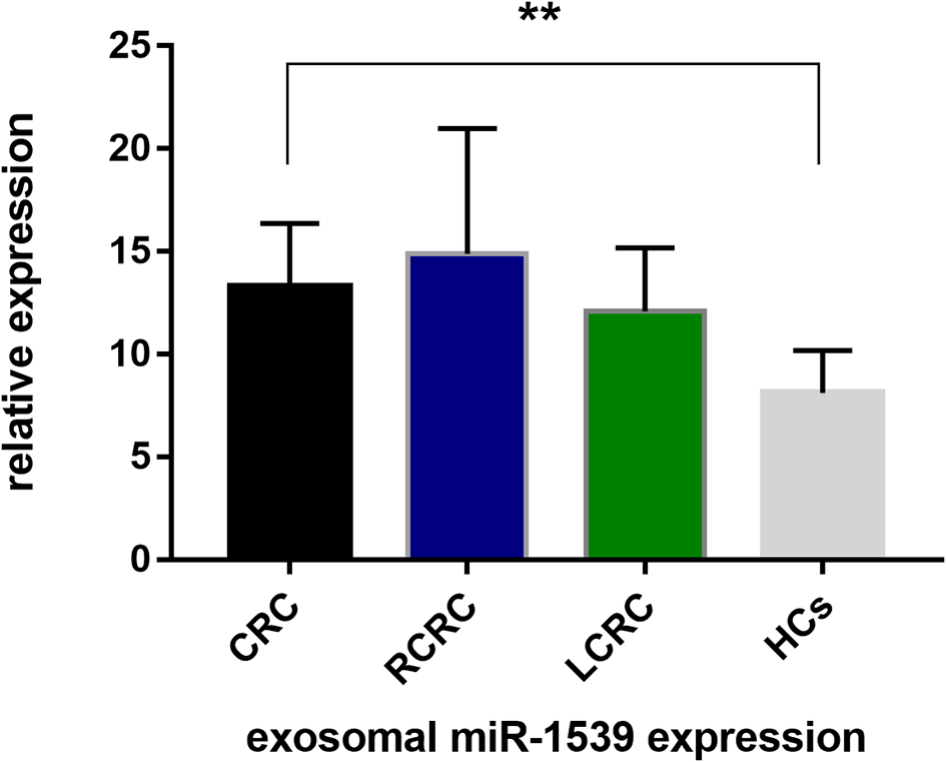
Exosome-based miR-1539 expression levels between CRC, LCRC, RCRC and HCs groups. Exosomal miR-1539 was upregulation in CRC (*p* = 0.003). Y-axis represents miR-1539 relative expression by 2^-△Ct^ method. Mean with 95%CI are performed in the bars. CRC, colorectal cancer; HCs, healthy controls. RCRC, right-sided CRC; LCRC, left-sided CRC. *p<0.05, **p<0.01

We analyzed exosome morphology using TEM. Our TEM images showed particles were cup-shaped, and within the anticipated size range for exosomes (**Figure 1A**). Particle sizes and concentrations were determined by NTA, and were on average, 102.9 nm in diameter (**Figure 1B**). The typical exosome markers, CD9 and TSG101 were detected by western blot, thereby authenticating the presence of exosomes (**Figure 1C**). These findings indicated that exosomal extraction methods were robust. The relative expression levels of exosomal miR-1539 were significantly elevated in CRC patients (*p* = 0.003) when compared with HCs (**Table 2**, **Figure 2**). We also analyzed the pathological parameters of CRC patients, however, no significant differences were observed in terms of tumor size, degree of pathological differentiation, lymphatic and distant metastasis, etc. **(** **Table 3**).

**Table 2:** Correlation between circulating miR-1539 expression levels in CRC and HCs.

	mean ± SD	*p* value (vs HC)
Exosome		
CRC	-3.31 ± 1.15	0.003**
RCRC	-3.43 ± 1.17	0.012*
LCRC	-3.22 ± 1.14	0.017*
HCs	-2.36 ± 1.61	
Serum		
CRC	-1.90 ± 1.48	0.104
RCRC	-2.32 ± 1.87	0.737
LCRC	-1.58 ± 1.03	0.031*
HCs	-2.72 ± 1.99	

CRC, colorectal cancer; RCRC, right-sided CRC; LCRC, left-sided CRC; HCs, healthy controls. *p < 0.05, **p < 0.01. Bold values represent p < 0.05.

**Table 3:** Correlation between clinicopathological factors and miR-1539 expression levels in exosome and serum.

	Exosome				Serum	
	n	mean ± SD	*p* value		mean ± SD	*p* value
Age(year)			0.947			0.449
≥ 60	23	-3.23 ± 1.31			-2.13 ± 1.72	
< 60	28	-3.38 ± 1.02			-1.71 ± 1.26	
Sex			0.235			0.568
Male	29	-3.13 ± 1.05			-1.76 ± 1.60	
Female	22	-3.55 ± 1.25			-2.09 ± 1.32	
Location			0.655			0.047*
RCRC	22	-3.43 ± 1.17			-2.32 ± 1.87	
LCRC	29	-3.22 ± 1.14			-1.58 ± 1.03	
Tumor size			0.189			0.515
≥ 5 cm	26	-3.45 ± 1.29			-2.07 ± 1.55	
< 5 cm	22	-2.99 ± 0.95			-1.66 ± 1.46	
Unknown	3					
Differentiation			0.512			0.93
Well and moderate	32	-3.19 ± 1.19			-1.95 ± 1.58	
Poor	16	-3.52 ± 1.12			-1.76 ± 1.40	
Unknown	3					
Lymphatic metastasis			0.992			0.517
Yes	28	-3.26 ± 1.16			-1.73 ± 1.35	
No	20	-3.36 ± 1.20			-2.09 ± 1.72	
Unknown	3					
Distant metastasis			0.699			0.725
Yes	11	-3.17 ± 1.54			-1.98 ± 1.06	
No	39	-3.32 ± 1.03			-1.85 ± 1.60	
Unknown	1					
Angiolymphatic invasion			0.371			0.992
Yes	18	-3.17 ± 0.88			-1.81 ± 1.28	
No	30	-3.39 ± 1.31			-1.93 ± 1.65	
Unknown	3					
Perineural invasion			0.857			0.448
Yes	47	-3.30 ± 1.18			-1.87 ± 1.52	
No	1	-3.32			-2.67	
Unknown	3					
Tumor deposits			0.624			0.915
Yes	3	-3.12 ± 1.39			-1.84 ± 0.75	
No	45	-3.32 ± 1.17			-1.89 ± 1.55	
Unknown	3					
TNM stage			0.576			0.712
I+II	19	-3.43 ± 1.18			-2.06 ± 1.76	
III+IV	31	-3.20 ± 1.13			-1.77 ± 1.32	
Unknown	1					
Ki-67			0.068			0.893
> 70%	11	-2.73 ± 1.17			-1.71 ± 1.38	
≤ 70%	37	-3.39 ± 1.12			-1.93 ± 1.56	
Unknown	3					
p53			0.498			0.361
negative	8	-3.32 ± 1.11			-1.30 ± 1.46	
positive	40	-3.22 ± 1.18			-2.00 ± 1.51	
Unknown	3					
EGFR			0.486			0.430
low	35	-3.32 ± 1.14			-1.88 ± 1.51	
high	13	-3.02 ± 1.20			-1.89 ± 1.56	
Unknown	3					
VEGF			0.136			0.028*
low	18	-3.40 ± 1.43			-1.30 ± 1.19	
high	30	-3.14 ± 0.97			-2.24 ± 1.58	
Unknown	3					

Bold values represent p < 0.05.

### Serum miR-1539 Expression Levels in CRC and Correlations With Pathological Parameters

**Figure 3:**
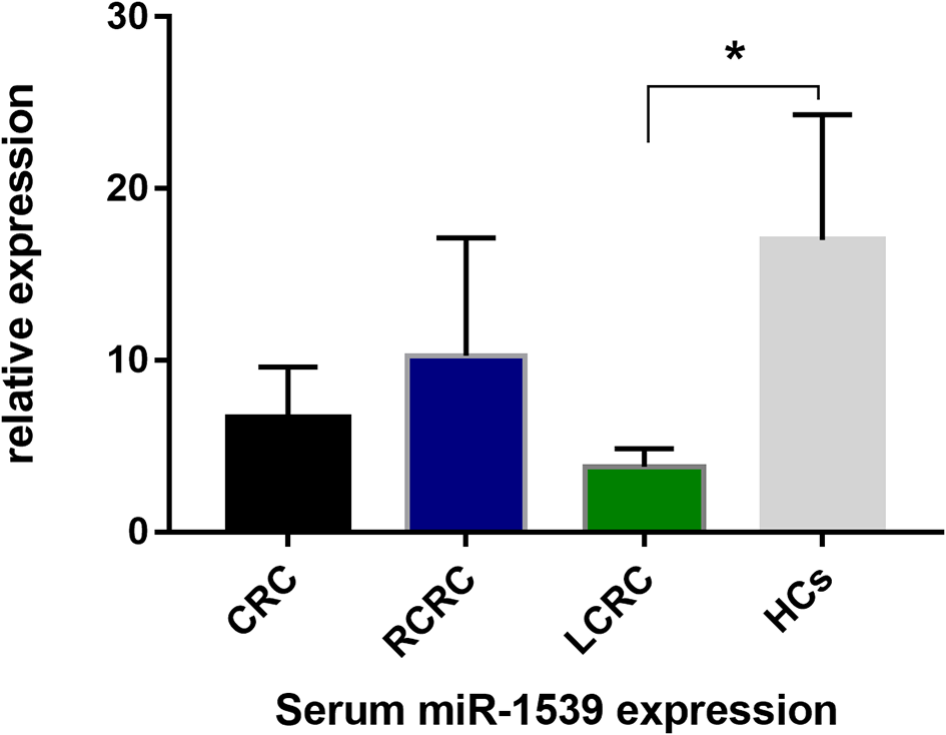
Serum-based miR-1539 expression levels between CRC, LCRC, RCRC, and HCs groups. The expression of serum miR-1539 decreased in LCRC (*p* = 0.031). Y-axis represents the miR-1539 relative expression by 2^-△Ct^ method. Mean with 95%CI are indicated in the bars. CRC, colorectal cancer; HCs, healthy controls; RCRC, right-sided colorectal cancer; LCRC, left-sided colorectal cancer. *p < 0.05.

We further explored serum miR-1539 expression to ascertain if levels were similarly upregulated as for exosomes. We observed no significant differences in miR-1539 expression levels between HCs and CRC cases (*p* = 0.104). However, we observed significant differences using stratified analyses by tumor sites. Serum miR-1539 expression levels were down-regulated in left-sided CRC (LCRC) when compared with HCs (*p* = 0.031) (**Table 2**, **Figure 3**). Similarly, differences in miR-1539 expression were also observed in terms of CRC tumor location. MiR-1539 was overexpressed in right-sided CRC (RCRC) when compared with LCRC (*p* = 0.047). Furthermore, VEGF expression in CRC samples was positively correlated with serum miR-1539 levels (*p* = 0.028) (**Table 3**).

### Diagnostic Efficacy of Circulating miR-1539 Using ROC Analysis

**Figure 4:**
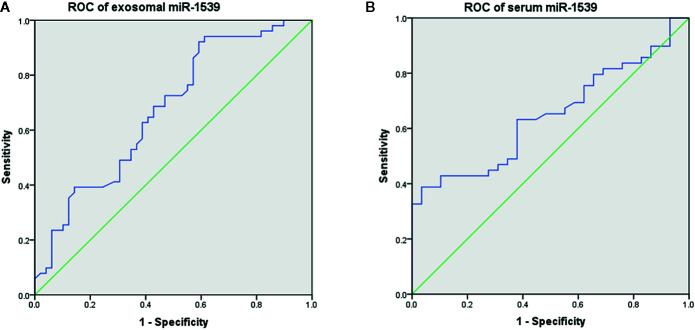
Diagnostic values of circulating miR-1539 based on ROC curves. **(A)** exosomal miR-1539 between CRC and HCs. **(B)** serum miR-1539 in LCRC and HCs. CRC, colorectal cancer; HCs, healthy controls; LCRC, left-sided colorectal cancer. *p < 0.05.

The diagnostic effects of circulating miR-1539 were investigated in exosomes and serum. ROC analyses showed that exosomal miR-1539 could distinguish CRC from HCs [AUC = 0.673, 95% confidence interval (CI) = 0.568–0.779, *p* = 0.003, **Figure 4A**], with a sensitivity of 92.2%, and specificity of 40.8%. Moreover, serum miR-1539 levels have the capability to differentiate LCRC from HCs (AUC = 0.647, 95%CI = 0.526–0.767, *p* = 0.031, **Figure 4B**), with a sensitivity and specificity of 38.8 and 96.6%, respectively.

### MiR-1539 Expression Levels in CRC Tissues and Correlations With Pathological Parameters

**Figure 5:**
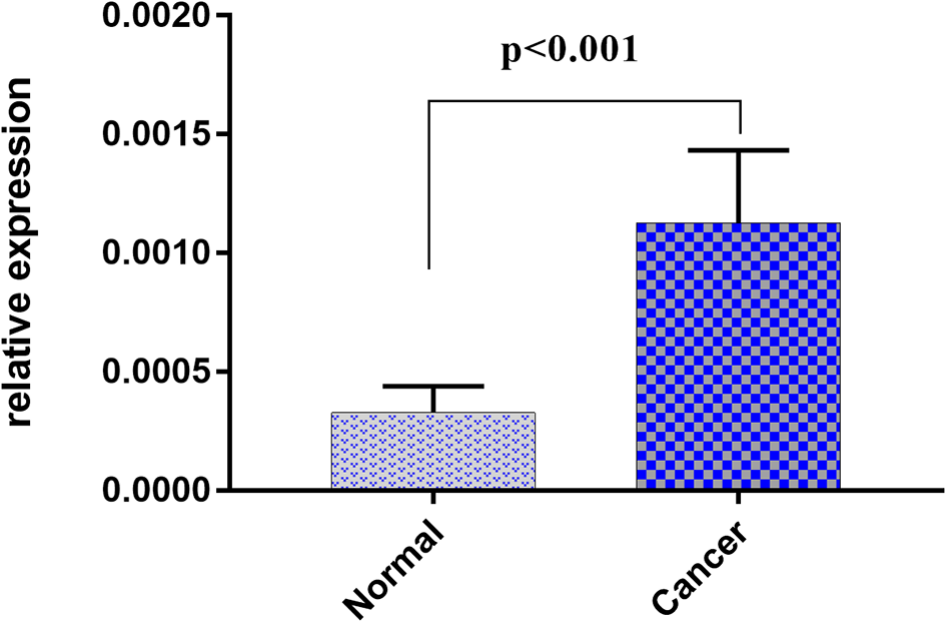
Relative expression of miR-1539 between cancer tissue versus matched normal tissue. miR-1539 was overexpression in cancer tissue. Y-axis represents miR-1539 relative expression by 2^-△Ct^ method. Mean with 95%CI are performed in the bars.

As circulating miR-1539 levels tended to vary, we investigated miR-1539 expression levels in CRC tissue and matched NAT samples using RT-qPCR. We observed significant miR-1539 up-regulation in CRC tissues when compared with NAT tissues (mean ± SD: 10.57 ± 1.80 vs. 12.55 ± 1.81, *p <*0.001) (**Figure 5**). Similarly, we also analyzed correlations between miR-1539 expression and clinicopathological parameters. Up-regulated miR-1539 was significantly associated with-elevated Ki-67 levels (*p* = 0.035). Additionally, miR-1539 expression had a growing tendency of poor pathologic differentiation (*p* = 0.071) (**Table 4**).

**Table 4:** Correlation between clinicopathological factors and miR-1539 expression levels in CRC tissues.

	n	mean ± SD	*p* value
Age(year)			0.389
≥ 60	29	10.75 ± 1.57	
< 60	27	10.49 ± 2.02	
Sex			0.168
Male	29	10.22 ± 1.31	
Female	27	11.06 ± 2.13	
Location			0.719
RCRC	18	10.43 ± 1.60	
LCRC	38	10.71 ± 1.89	
Tumor size			0.967
≥ 5cm	31	10.74 ± 2.11	
< 5cm	25	10.47 ± 1.31	
Differentiation			0.071
Well and moderate	42	10.87 ± 1.83	
Poor	14	9.88 ± 1.49	
Lymphatic metastasis			0.172
Yes	26	10.14 ± 1.50	
No	29	10.99 ± 1.95	
Unknown	1		
Distant metastasis			0.664
Yes	9	11.04 ± 2.35	
No	47	10.54 ± 1.68	
Angiolymphatic invasion			0.565
Yes	21	10.47 ± 1.83	
No	35	10.71 ± 1.78	
Perineural invasion			0.171
Yes	52	10.55 ± 1.79	
No	4	11.61 ± 1.62	
TNM stage			0.237
I+II	26	10.35 ± 1.76	
III+IV	30	10.94 ± 1.80	
Ki-67			0.035*
> 70%	16	9.79 ± 1.27	
≤ 70%	40	10.95 ± 1.87	
p53			0.751
negative	16	10.55 ± 1.69	
positive	40	10.65 ± 1.85	
EGFR			0.732
low	15	10.58 ± 1.74	
high	41	10.64 ± 1.83	
VEGF			0.541
low	13	10.74 ± 1.91	
high	43	10.24 ± 1.32	

Bold values represent p < 0.05.

### MiR-1539 Target Gene Predictions Using Bioinformatics, and Subsequent Validation by RT-qPCR

**Figure 6:**
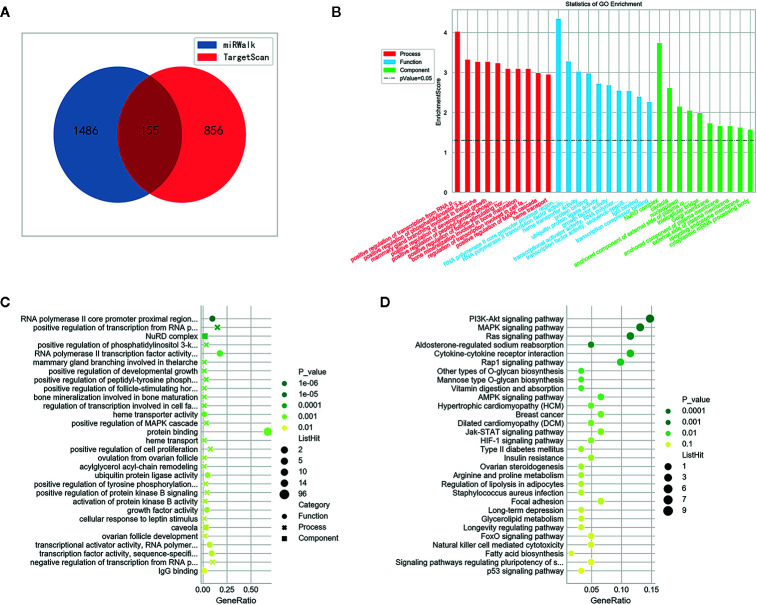
Bioinformatics analysis of miR-1539. **(A)** 155 potential genes were discovered by intersection of target genes prediction from miRWalk and TargetScan datasets. **(B)** GO enrichment: Potential target genes were classified by three functional categories: biological process, molecular functions, and cellular component (p < 0.05). TOP 10 potential target genes of each item were displayed in the bar plot. **(C)** GO enrichment: bubble plot showed TOP 30 most significant items of potential target genes in all functional categories for miR-1539 (p < 0.05). The top significantly target genes and scores were listed as y-axis and x-axis, respectively. **(D)** KEGG pathway analysis indicated that miR-1539 was observably enriched in cancer signaling pathway, using the DAVID. The enriched pathways and scores were listed as y-axis and x-axis, respectively.

We identified potential target genes of miR-1539 using an integrated analysis of miRWalk and TargetScan datasets. In total, 155 potential genes were identified using synthesis analysis of public bioinformatics algorithms (**Figure 6A**). GO analyses demonstrated that the majority of miR-1539 target genes were enriched for biological processes including, positive regulation of transcription, developmental growth and cell proliferation, and molecular functions such as RNA polymerase II transcription factor activity, and growth factor activity, etc. (**Figures 6B, C**). KEGG enrichment analyses revealed that miR-1539 was involved in several cancer signaling pathways, including, phosphatidylinositol 3-kinase (PI3K), mitogen activated protein kinase (MAPK), the janus kinase (JAK)-signal transducer and activator of transcription (STAT), Ras, receptor-associated protein-1 (Rap-1), adenosine 5’-monophosphate activated protein kinase (AMPK), hypoxia inducible factor (HIF) and p53 (**Figure 6D**).

Further target gene investigations were conducted with CRC tissue and matched NAT, using RT-qPCR. We randomly screened nine candidate target genes based on bioinformatics. Differences in expression were identified (**Supplemental Table 2**), of which GPIHBP1 and IGF1 were significantly down-regulated in CRC tissues (both *p* values < 0.001). Differentially up-regulated genes included GJA4 (*p* = 0.003), ARFGAP (*p* < 0.001), ZHX3 (*p* = 0.008), and FLT1 (*p* = 0.004) in CRC tissue samples (**Supplemental Figure 1**).

## Discussion

In this study, the relationship between miR-1539 expression levels and CRC risk and clinical parameters was confirmed using exosome, serum, and tissue assays. We also evaluated the diagnostic efficacy of miR-1539 towards CRC using ROC curve analysis. Furthermore, target genes of miR-1539 and their functions were analyzed using bioinformatics approaches. To our knowledge, this is the first study that has identified correlations between miR-1539 expression and CRC risk and clinicopathological biological behavior, based on a multidimensional-assay approach.

Liquid biopsy is a novel emerging molecular diagnostic technique, where exosomal dissection is performed in an accessible and timely manner to provide vital information in clinical settings (7, 22). Exosomes are small extracellular, 30–100 nm diameter vesicles, secreted by a variety of cells into body fluids including the blood, urine, cerebrospinal fluid, and exudates (23, 24). Derived from endosomes, exosomes carry unique cargo such as RNA (typically miRNA), proteins, and lipids. Exosomes travel to adjacent and distant target cells, inducing intercellular communications and gene regulation (23, 25, 26). With an inherent ability to actively select and enrich for miRNAs, tumor-derived exosomes transfer specific cell-independent mature miRNAs to recipient cells, to change their biological function, to silence mRNAs and promote oncogenesis (27, 28). Currently, circulating miRNAs are believed to originate from exosomes, and are regarded as potential biomarkers for the diagnosis of various cancers (22, 29, 30). In this study, exosomal miR-1539 from CRC patients was highly expressed, suggesting this miR could predict CRC risk, or participate in CRC pathological processes. ROC curve analyses showed that the sensitivity of exosomal miR-1539 was approximately 92.2%, which indicated it efficiently distinguished CRC from HC samples. Hence, exosomal miR-1539 may be used as a non-invasive and sensitive biomarker in clinical practice to screen high-risk CRC populations.

We also investigated miR-1539 expression levels in serum. Interestingly, we observed no statistically significant differences in serum miR-1539 expression levels between CRC and HCs samples. However, MiR-1539 expression in serum was decreased in LCRC with a specificity of 96.6%, when compared with HCs, suggesting miR-1539 may be an indicator of LCRC. However, this result was not consistent with exosomes. Unlike the active selection of miRNAs in exosomes where miRNA molecules are selectively enriched, numerous cell-free miRNAs are passively released into the circulation during necrosis and apoptosis mechanisms. Their release from interacting with Argonaute (AGO) proteins which were recruited by target RNAs, may not reflect dynamic cancer responses (31, 32). This observation could explain the miR-1539 differential expression we observed in exosomes and serum. Another serum based miR-1539 expression inconsistency was the variation in tumor location. Previous studies have identified distinctions between RCRC and LCRC in terms of epidemiology, molecular mechanisms, prognostics and therapeutic regimens (33–35). Therefore, primary location may be a critical independent prognostic factor, especially for untreated metastatic CRC (33, 36). In our study, serum based miR-1539 was positively correlated with VEGF expression (*p* = 0.028). As an essential angiogenic regulator, VEGF contributes to cancer genesis and development, but is also a vital therapeutic target (37). It has been suggested that up-regulation of VEGF is associated with advanced cancer stages, particularly lymph node metastasis and angiolymphatic invasion, and it may also predict a poor prognosis for CRC (38, 39).Taken together, serum based miR-1539 may provide clues underpinning molecular mechanisms for discrepant CRC prognoses (RCRC versus LCRC), and targeted anti-VEGF therapy. However, further studies are required to verify these observations.

Tissue biopsy is a gold standard approach used in clinical settings to identify tumor expression profiles. After the integrated analysis of circulating miR-1539, miR-1539 expression levels in cancer tissues were also examined. MiR-1539 had higher expression levels in CRC when compared to NAT tissue. From these observations, we inferred that miR-1539 up-regulation in tissue could be a CRC warning sign, thus exemplifying its role in CRC carcinogenesis. Combined with clinicopathological parameters, miR-1539 overexpression was also associated with elevated Ki-67 expression levels. Ki-67 is a well-accepted marker of tumor cell proliferation; it is detected in almost all proliferative phases of the cell cycle, except for the stationary stage (40). Several studies have confirmed that high Ki-67 levels are associated with a poor CRC prognosis (41–43), suggesting miR-1539 may also be linked to poor CRC outcomes. In terms of other pathological factors, miR-1539 overexpression displayed a correlation tendency with poor pathological differentiation, although we observed no statistical significance for this (*p* = 0.071). Overall, tissue based miR-1539 may not only predict CRC onset, but also a poor CRC prognosis. However, further studies must be conducted in larger cohorts.

Given miR-1539 overexpression in both exosomes and cancer tissue, we conducted a bioinformatics analysis to explore potential regulatory mechanisms of miR-1539 in CRC. GO annotation analysis showed that miR-1539 may bind to sequences in the proximal region of the RNA polymerase II (Pol II) core, and positively modulate transcription from the Pol II promoter. Such actions could culminate in cancer mechanisms; e.g., dysregulation of transcriptional elongation mediated by Pol II leads to cancer (44). MiR-1539 was also implicated in several cancer signaling pathways, including PI3K-Akt, MAPK, Ras, Jak-STAT, and p53, which have been implicated in CRC initiation (45–49). These findings suggest that miR-1539 may mechanistically influence CRC genesis and progression. Further, we randomly screened nine candidate target genes based on bioinformatics. The results showed that GPIHBP1 and IGF1 were significantly down-regulated in CRC tissues while GJA4, ARFGAP, ZHX3, and FLT1 were significantly up-regulated in CRC tissue. As known, miRNAs suppress or even silence target genes by binding to complementary 3’ untranslated regions (3’UTRs) (5). In our study, miR-1539 was overexpression in cancerous tissue, we considered GPIHBP1, IGF1 were potential target gene, due to the principle of miRNAs negative mediation of target genes.

Our study had several limitations. Based on the existing literature, there is no “gold standard” exosomal miRNA or ideal endogenous molecule for RT-qPCR normalization in body fluids; some studies recommend spiked-in controls, i.e., *Caenorhabditis elegans* (cel)-miR-39 or cel-miR-54 (50, 51). As a classical internal control, U6 is still used to normalize miRNA expression levels in blood or other fluids (52–54). In our study, we used U6 as an internal control, without spiked-in controls. Thus, in the future, large-scale testing and verification of internal controls is required. Equally, to comprehensively elucidate the role and mechanism of miR-1539 in CRC tumorigenesis and progression, more large-cohort investigations should be conducted.

In conclusion, we investigated miR-1539 expression levels in exosomes, serum, and tissue, and observed that miR-1539 was up-regulated in exosomes and CRC tissue. In terms of diagnostic efficacy, exosome based miR-1539 may help distinguish CRC cases from HCs, and specificity for LCRC may be improved by analyzing serum miR-1539 levels. Additionally, a positive miR-1539 tissue biopsy could be treated as a warning sign for CRC. The combined assessment of serum miR-1539 levels may enhance the diagnostic efficacy for CRC, especially LCRC. In terms of pathological parameters, serum miR-1539 appeared to be positively associated with VEGF expression, and CRC tumor location. In tissue, elevated miR-1539 detection may confer a poor CRC prognosis if increased Ki-67 levels are concomitantly observed. The multidimensional detection of circulating or tissue based miR-1539 could serve as a novel biomarker for CRC early screening. Moreover, miR-1539 could provide insights on CRC pathogenesis owing to its predictive abilities towards tumor sites and poor clinicopathological behavior. Comprehensive, multicenter validation studies are warranted in the future.

## Data Availability Statement

All datasets generated for this study are included in the article/**Supplementary Material**.

## Ethics Statement

The studies involving human participants were reviewed and approved by ethics committee of the First Hospital of China Medical University. The patients/participants provided their written informed consent to participate in this study. Written informed consent was obtained from the individual(s) for the publication of any potentially identifiable images or data included in this article.

## Author Contributions

YY contributed study design and revising the manuscript. XC contributed sample collection, data interpretation, performing the experiment, and drafting manuscript. ZL, HD, and CX partly contributed sample collection, data interpretation and manuscript embellishment. All authors contributed to the article and approved the submitted version.

## Funding

This study was supported by the National Key R&D Program of China (Grant #2018YFC1311600) and Liaoning Revitalization Talents Program (Grant #XLYC1808036).

## Conflict of Interest

The authors declare that the research was conducted in the absence of any commercial or financial relationships that could be construed as a potential conflict of interest.

## Supplementary Material

Supplementary Figure 1 Validation of candidate target gene in CRC patients’ samples by RT-qPCR **(A, B)** Significant downregulation of GPIHBP1 and IGF1 were observed in cancerous tissue of CRC samples. **(C)** Four genes overexpressed in CRC tissue. Y-axis interprets the candidate gene relative expression by 2^-△Ct^ method. Mean with SEM are displayed in the bars. (*p < 0.05, **p < 0.01, *p < 0.001).

## References

[1] Bray F Ferlay J Soerjomataram I Siegel RL Torre LA Jemal A. Global cancer statistics 2018: GLOBOCAN estimates of incidence and mortality worldwide for 36 cancers in 185 countries. CA Cancer J Clin (2018) 68:394–424. 10.3322/caac.21492 30207593

[2] Arnold M Sierra MS Laversanne M Soerjomataram I Jemal A Bray F. Global patterns and trends in colorectal cancer incidence and mortality. Gut (2017) 66:683–91. 10.1136/gutjnl-2015-310912 26818619

[3] Lee RC Feinbaum RL Ambros V. The C. elegans heterochronic gene lin-4 encodes small RNAs with antisense complementarity to lin-14. Cell (1993) 75:843–54. 10.1016/0092-8674(93)90529-y 8252621

[4] Abramowicz A Story M. The Long and Short of It: The Emerging Roles of Non-Coding RNA in Small Extracellular Vesicles. Cancers (2020) 12:1445. 10.3390/cancers12061445 PMC7352322 32498257

[5] Gebert L MacRae I. Regulation of microRNA function in animals. Nat Rev Mol Cell Biol (2019) 20:21–37. 10.1038/s41580-018-0045-7 30108335 PMC6546304

[6] Kim S Jeon O Jeon Y. Extracellular RNA: Emerging roles in cancer cell communication and biomarkers. Cancer Lett (2020) 495:33–40. 10.1016/j.canlet.2020.09.002 32916182

[7] Bottani M Banfi G Lombardi G. Circulating miRNAs as Diagnostic and Prognostic Biomarkers in Common Solid Tumors: Focus on Lung, Breast, Prostate Cancers, and Osteosarcoma. J Clin Med (2019) 8:1661. 10.3390/jcm8101661 PMC6833074 31614612

[8] Wang J Ni J Beretov J Thompson J Graham P Li Y. Exosomal microRNAs as liquid biopsy biomarkers in prostate cancer. Crit Rev Oncol/Hematol (2020) 145:102860. 10.1016/j.critrevonc.2019.102860 31874447

[9] Cui C Cui Q. The relationship of human tissue microRNAs with those from body fluids. Sci Rep (2020) 10:5644. 10.1038/s41598-020-62534-6 32221351 PMC7101318

[10] Jayaseelan V. Emerging role of exosomes as promising diagnostic tool for cancer. Cancer Gene Ther (2020) 27:395–8. 10.1038/s41417-019-0136-4 31477807

[11] Pang B Zhu Y Ni J Thompson J Malouf D Bucci J. Extracellular vesicles: the next generation of biomarkers for liquid biopsy-based prostate cancer diagnosis. Theranostics (2020) 10:2309–26. 10.7150/thno.39486 PMC7019149 32089744

[12] Cui M Wang H Yao X Zhang D Xie Y Cui R. Circulating MicroRNAs in Cancer: Potential and Challenge. Front Genet (2019) 10:626. 10.3389/fgene.2019.00626 31379918 PMC6656856

[13] De Palma F Luglio G Tropeano F Pagano G D’Armiento M Kroemer G. The Role of Micro-RNAs and Circulating Tumor Markers as Predictors of Response to Neoadjuvant Therapy in Locally Advanced Rectal Cancer. Int J Mol Sci (2020) 21:7040. 10.3390/ijms21197040 PMC7582560 32987896

[14] Yang X Zhang Q Zhang M Su W Wang Z Li Y. Serum microRNA Signature Is Capable of Early Diagnosis for Non-Small Cell Lung Cancer. Int J Biol Sci (2019) 15:1712–22. 10.7150/ijbs.33986 PMC6643220 31360113

[15] Marcuello M Vymetalkova V Neves R Duran-Sanchon S Vedeld H Tham E. Circulating biomarkers for early detection and clinical management of colorectal cancer. Mol Asp Med (2019) 69:107–22. 10.1016/j.mam.2019.06.002 31189073

[16] Mahdiannasser M Karami Z. An innovative paradigm of methods in microRNAs detection: highlighting DNAzymes, the illuminators. Biosens Bioelectron (2018) 107:123–44. 10.1016/j.bios.2018.02.020 29455023

[17] Dave V Ngo T Pernestig A Tilevik D Kant K Nguyen T. MicroRNA amplification and detection technologies: opportunities and challenges for point of care diagnostics. Lab Investig J Tech Methods Pathol (2019) 99:452–69. 10.1038/s41374-018-0143-3 30542067

[18] He T Sun R Li Y Katusic ZS. Effects of Brain-Derived Neurotrophic Factor on MicroRNA Expression Profile in Human Endothelial Progenitor Cells. Cell Transplant (2018) 27:1005–9. 10.1177/0963689718761658 PMC6050915 29860902

[19] Ayaz L Dinc E. Evaluation of microRNA responses in ARPE-19 cells against the oxidative stress. Cutan Ocul Toxicol (2018) 37:121–6. 10.1080/15569527.2017.1355314 28707489

[20] Veija T Sahi H Koljonen V Bohling T Knuutila S Mosakhani N. miRNA-34a underexpressed in Merkel cell polyomavirus-negative Merkel cell carcinoma. Virchows Arch (2015) 466:289–95. 10.1007/s00428-014-1700-9 25491743

[21] Mi H Huang X Muruganujan A Tang H Mills C Kang D. PANTHER version 11: expanded annotation data from Gene Ontology and Reactome pathways, and data analysis tool enhancements. Nucleic Acids Res (2017) 45:D183–d9. 10.1093/nar/gkw1138 PMC5210595 27899595

[22] Shi Y Wang Z Zhu X Chen L Ma Y Wang J. Exosomal miR-1246 in serum as a potential biomarker for early diagnosis of gastric cancer. Int J Clin Oncol (2020) 25:89–99. 10.1007/s10147-019-01532-9 31506750

[23] Kourembanas S. Exosomes: vehicles of intercellular signaling, biomarkers, and vectors of cell therapy. Annu Rev Physiol (2015) 77:13–27. 10.1146/annurev-physiol-021014-071641 25293529

[24] Min L Zhu S Chen L Liu X Wei R Zhao L. Evaluation of circulating small extracellular vesicles derived miRNAs as biomarkers of early colon cancer: a comparison with plasma total miRNAs. J Extracell Vesicles (2019) 8:1643670. 10.1080/20013078.2019.1643670 31448068 PMC6691764

[25] Milane L Singh A Mattheolabakis G Suresh M Amiji MM. Exosome mediated communication within the tumor microenvironment. J Control Release (2015) 219:278–94. 10.1016/j.jconrel.2015.06.029 26143224

[26] Guo S Chen J Chen F Zeng Q Liu W Zhang G. Fusobacterium nucleatumExosomes derived from -infected colorectal cancer cells facilitate tumour metastasis by selectively carrying miR-1246/92b-3p/27a-3p and CXCL16. Gut (2020) 1–13. 10.1136/gutjnl-2020-321187 33172926

[27] Manier S Liu C Avet-Loiseau H Park J Shi J Campigotto F. Prognostic role of circulating exosomal miRNAs in multiple myeloma. Blood (2017) 129:2429–36. 10.1182/blood-2016-09-742296 PMC5409448 28213378

[28] Shi Z Yang X Malichewe C Li Y Guo X. Exosomal microRNAs-mediated intercellular communication and exosome-based cancer treatment. Int J Biol Macromol (2020) 158:530–41. 10.1016/j.ijbiomac.2020.04.228 32360962

[29] Zhang Y Zhang Y Yin Y Li S. Detection of circulating exosomal miR-17-5p serves as a novel non-invasive diagnostic marker for non-small cell lung cancer patients. Pathol Res Pract (2019) 215:152466. 10.1016/j.prp.2019.152466 31146974

[30] Kim S Choi MC Jeong JY Hwang S Jung SG Joo WD. Serum exosomal miRNA-145 and miRNA-200c as promising biomarkers for preoperative diagnosis of ovarian carcinomas. J Cancer (2019) 10:1958–67. 10.7150/jca.30231 PMC6548168 31205555

[31] Fabris L Calin G. Circulating free xeno-microRNAs - The new kids on the block. Mol Oncol (2016) 10:503–8. 10.1016/j.molonc.2016.01.005 PMC4880025 26860056

[32] Fernández-Lázaro D García Hernández J García A Córdova Martínez A Mielgo-Ayuso J Cruz-Hernández J. Liquid Biopsy as Novel Tool in Precision Medicine: Origins, Properties, Identification and Clinical Perspective of Cancer’s Biomarkers. Diagn (Basel Switzerland) (2020) 10:215. 10.3390/diagnostics10040215 PMC7235853 32294884

[33] Zheng C Jiang F Lin H Li S. Clinical characteristics and prognosis of different primary tumor location in colorectal cancer: a population-based cohort study. Clin Trans Oncol Off Publ Fed Spanish Oncol Soc Natl Cancer Inst Mexico (2019) 21:1524–31. 10.1007/s12094-019-02083-1 30875062

[34] Kim K Kim YW Shim H Kim BR Kwon HY. Differences in clinical features and oncologic outcomes between metastatic right and left colon cancer. J Buon (2018) 23:11–8. 30722106

[35] Siegel RL Miller KD Fedewa SA Ahnen DJ Meester RGS Barzi A. Colorectal cancer statistics, 2017. CA Cancer J Clin (2017) 67:177–93. 10.3322/caac.21395 28248415

[36] Stintzing S Tejpar S Gibbs P Thiebach L Lenz H. Understanding the role of primary tumour localisation in colorectal cancer treatment and outcomes. Eur J Cancer (Oxford Engl 1990) (2017) 84:69–80. 10.1016/j.ejca.2017.07.016 PMC7505124 28787661

[37] Melincovici C Boşca A Şuşman S Mărginean M Mihu C Istrate M. Vascular endothelial growth factor (VEGF) - key factor in normal and pathological angiogenesis. Rom J Morphol Embryol = Rev Roum Morphol Embryol (2018) 59:455–67. 30173249

[38] Mazeda I Martins SF Garcia EA Rodrigues M Longatto A. VEGF expression in colorectal cancer metastatic lymph nodes: clinicopathological correlation and prognostic significance. Gastrointest Disord (2020) 2:267–80. 10.3390/gidisord2030025

[39] Mohamed SY Mohammed HL Ibrahim HM Mohamed EM Salah M. Role of VEGF, CD105, and CD31 in the Prognosis of Colorectal Cancer Cases. J Gastrointest Cancer (2019) 50:23–34. 10.1007/s12029-017-0014-y 29110224

[40] Rödel F Zhou S Győrffy B Raab M Sanhaji M Mandal R. The Prognostic Relevance of the Proliferation Markers Ki-67 and Plk1 in Early-Stage Ovarian Cancer Patients With Serous, Low-Grade Carcinoma Based on mRNA and Protein Expression. Front Oncol (2020) 10:558932. 10.3389/fonc.2020.558932 33117692 PMC7577119

[41] Yang Y Li J Jin L Wang D Zhang J Wang J. Independent Correlation Between Ki67 Index and Circulating Tumor Cells in the Diagnosis of Colorectal Cancer. Anticancer Res (2017) 37:4693–700. 10.21873/anticanres.11874 28739773

[42] Wang L Liu Z Fisher K Ren F Lv J Davidson D. Prognostic value of programmed death ligand 1, p53, and Ki-67 in patients with advanced-stage colorectal cancer. Hum Pathol (2018) 71:20–9. 10.1016/j.humpath.2017.07.014 28782638

[43] Luo Z Zhu M Zhang Z Ye F Huang W Luo X. Increased expression of Ki-67 is a poor prognostic marker for colorectal cancer patients: a meta analysis. BMC Cancer (2019) 19:123. 10.1186/s12885-019-5324-y 30727976 PMC6364416

[44] Chen F Smith E Shilatifard A. Born to run: control of transcription elongation by RNA polymerase II. Nat Rev Mol Cell Biol (2018) 19:464–78. 10.1038/s41580-018-0010-5 29740129

[45] Zhang Y Kwok-Shing Ng P Kucherlapati M Chen F Liu Y Tsang Y. A Pan-Cancer Proteogenomic Atlas of PI3K/AKT/mTOR Pathway Alterations. Cancer Cell (2017) 31:820–32.e3. 10.1016/j.ccell.2017.04.013 28528867 PMC5502825

[46] Li Y Zeng C Hu J Pan Y Shan Y Liu B. Long non-coding RNA-SNHG7 acts as a target of miR-34a to increase GALNT7 level and regulate PI3K/Akt/mTOR pathway in colorectal cancer progression. J Hematol Oncol (2018) 11:89. 10.1186/s13045-018-0632-2 29970122 PMC6029165

[47] Jiang L Zhao X Mao Y Wang J Zheng H You Q. Long non-coding RNA RP11-468E2.5 curtails colorectal cancer cell proliferation and stimulates apoptosis via the JAK/STAT signaling pathway by targeting STAT5 and STAT6. J Exp Clin Cancer Res CR (2019) 38:465. 10.1186/s13046-019-1428-0 31718693 PMC6852742

[48] Wu Q Deng J Fan D Duan Z Zhu C Fu R. Ginsenoside Rh4 induces apoptosis and autophagic cell death through activation of the ROS/JNK/p53 pathway in colorectal cancer cells. Biochem Pharmacol (2018) 148:64–74. 10.1016/j.bcp.2017.12.004 29225132

[49] Bahrami A Hassanian S ShahidSales S Farjami Z Hasanzadeh M Anvari K. Targeting RAS signaling pathway as a potential therapeutic target in the treatment of colorectal cancer. J Cell Physiol (2018) 233:2058–66. 10.1002/jcp.25890 28262927

[50] Schlosser K Taha M Stewart D. Systematic Assessment of Strategies for Lung-targeted Delivery of MicroRNA Mimics. Theranostics (2018) 8:1213–26. 10.7150/thno.22912 PMC5835931 29507615

[51] Figueredo D Barbosa M Coimbra D Dos Santos J Costa E Koike B. Usual normalization strategies for gene expression studies impair the detection and analysis of circadian patterns. Chronobiol Int (2018) 35:378–91. 10.1080/07420528.2017.1410168 29219623

[52] He Z Li W Zheng T Liu D Zhao S. Human umbilical cord mesenchymal stem cells-derived exosomes deliver microRNA-375 to downregulate ENAH and thus retard esophageal squamous cell carcinoma progression. J Exp Clin Cancer Res CR (2020) 39:140. 10.1186/s13046-020-01631-w 32698859 PMC7374920

[53] Liu S Lin Z Zheng Z Rao W Lin Y Chen H. Serum exosomal microRNA-766-3p expression is associated with poor prognosis of esophageal squamous cell carcinoma. Cancer Sci (2020) 111:3881–92. 10.1111/cas.14550 PMC7540979 32589328

[54] Santangelo A Imbrucè P Gardenghi B Belli L Agushi R Tamanini A. A microRNA signature from serum exosomes of patients with glioma as complementary diagnostic biomarker. J Neuro-Oncol (2018) 136:51–62. 10.1007/s11060-017-2639-x 29076001

