# Supplementary figures and images for: MiR-1539 and Its Potential Role as a Novel Biomarker for Colorectal Cancer
Source: Front Oncol. 2021 Feb 18;10:531244. doi: 10.3389/fonc.2020.531244 (PMC7930495; doi:10.3389/fonc.2020.531244)

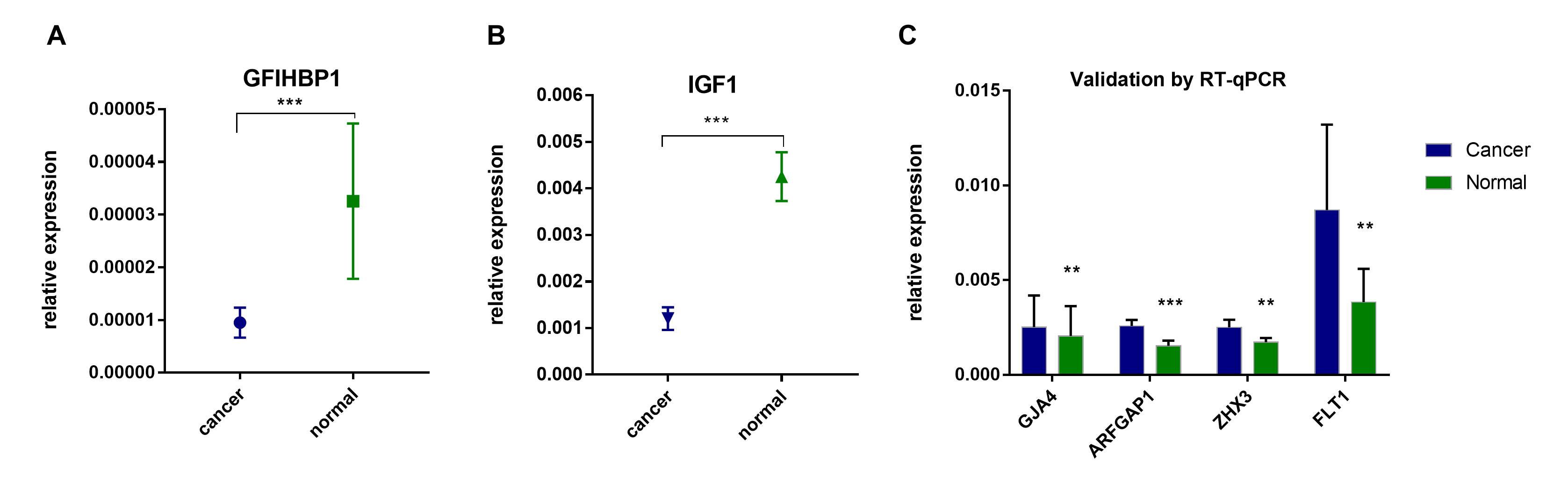

Supplement: Supplementary Figure 1 — Validation of candidate target gene in CRC patients’ samples by RT-qPCR (A, B) Significant downregulation of GPIHBP1 and IGF1 were observed in cancerous tissue of CRC samples. (C) Four genes overexpressed in CRC tissue. Y-axis interprets the candidate gene relative expression by 2-△Ct method. Mean with SEM are displayed in the bars. (*p < 0.05, **p < 0.01, *p < 0.001). [file Image_1.tif]
